# Preventive interventions to improve older people’s health outcomes: systematic review and meta-analysis

**DOI:** 10.3399/BJGP.2023.0180

**Published:** 2024-03-19

**Authors:** Leah Palapar, Jeanet W Blom, Laura Wilkinson-Meyers, Thomas Lumley, Ngaire Kerse

**Affiliations:** Department of General Practice and Primary Health Care, Faculty of Medical and Health Sciences, University of Auckland, Auckland, New Zealand.; Department of Public Health and Primary Care, Leiden University Medical Center, Leiden, the Netherlands.; Faculty of Medical and Health Sciences, University of Auckland, Auckland, New Zealand.; Department of Statistics, Faculty of Science, University of Auckland, Auckland, New Zealand.; Department of General Practice and Primary Health Care, Faculty of Medical and Health Sciences, University of Auckland, Auckland, New Zealand.

**Keywords:** aged, aged, 80 and over, general practice, healthcare use, patient reported outcome measures, activities of daily living

## Abstract

**Background:**

Systematic reviews of preventive, non-disease-specific primary care trials for older people often report effects according to what is thought to be the intervention’s active ingredient.

**Aim:**

To examine the effectiveness of preventive primary care interventions for older people and to identify common components that contribute to intervention success.

**Design and setting:**

A systematic review and meta-analysis of 18 randomised controlled trials (RCTs) published in 22 publications from 2009 to 2019.

**Method:**

A search was conducted in PubMed, MEDLINE, Embase, Web of Science, CENTRAL, CINAHL, and the Cochrane Library. Inclusion criteria were: sample mainly aged ≥65 years; delivered in primary care; and non-disease-specific interventions. Exclusion criteria were: non-RCTs; primarily pharmacological or psychological interventions; and where outcomes of interest were not reported. Risk of bias was assessed using the original Cochrane tool. Outcomes examined were healthcare use including admissions to hospital and aged residential care (ARC), and patient-reported outcomes including activities of daily living (ADLs) and self-rated health (SRH).

**Results:**

Many studies had a mix of patient-, provider-, and practice-focused intervention components (13 of 18 studies). Studies included in the review had low-to-moderate risk of bias. Interventions had no overall benefit to healthcare use (including admissions to hospital and ARC) but higher basic ADL scores were observed (standardised mean difference [SMD] 0.21, 95% confidence interval [CI] = 0.01 to 0.40) and higher odds of reporting positive SRH (odds ratio [OR] 1.17, 95% CI = 1.01 to 1.37). When intervention effects were examined by components, better patient-reported outcomes were observed in studies that changed the care setting (SMD for basic ADLs 0.21, 95% CI = 0.01 to 0.40; OR for positive SRH 1.17, 95% CI = 1.01 to 1.37), included educational components for health professionals (SMD for basic ADLs 0.21, 95% CI = 0.01 to 0.40; OR for positive SRH 1.27, 95% CI = 1.05 to 1.55), and provided patient education (SMD for basic ADLs 0.28, 95% CI = 0.09 to 0.48). Additionally, admissions to hospital in intervention participants were fewer by 23% in studies that changed the care setting (incidence rate ratio [IRR] 0.77, 95% CI = 0.63 to 0.95) and by 26% in studies that provided patient education (IRR 0.74, 95% CI = 0.56 to 0.97).

**Conclusion:**

Preventive primary care interventions are beneficial to older people’s functional ability and SRH but not other outcomes. To improve primary care for older people, future programmes should consider delivering care in alternative settings, for example, home visits and phone contacts, and providing education to patients and health professionals as these may contribute to positive outcomes.

## Introduction

Reliable research evidence is one of the key elements of evidence-based decision making.^1^ With multimorbidity on the rise, particularly for older people,^2^^,^^3^ the need to generate relevant evidence to guide management of older primary care patients^4^^–^^6^ and the use of broader outcome measures that matter to older people,^5^^–^^7^ such as maintaining independence and quality of life (QoL), is increasingly emphasised in the literature.

Many intervention trials set in primary care have tested the effectiveness of preventive rather than disease-specific approaches relating to the care of older people, and numerous systematic reviews related to this topic have been published. Previous reviews have found mixed effects on older people’s admissions to hospital^8^^–^^13^ and aged residential care (ARC),^8^^–^^10^^,^^12^^,^^13^ functional ability,^8^^,^^9^^,^^12^^–^^16^ and QoL.^11^^–^^14^ These reviews often report effects according to the primary intervention focus or what is thought to be its active ingredient — for example, multidimensional assessment and subsequent management;^8^^,^^9^^,^^14^^,^^17^ health promotion and disease prevention programmes;^15^^,^^16^^,^^18^ or preventive home visiting.^19^^,^^20^ However, preventive strategies and intervention programmes typically include multiple intervention components to address diverse objectives for change. The most recent Medical Research Council (MRC) guidance^21^ emphasises how complexity may arise from the content of interventions (for example, having several potentially interacting intervention components and mechanisms of change) or the context within which the intervention is implemented.

**Table:** How this fits in

Many primary care trials have tested the effectiveness of preventive, non-disease-specific approaches to older people’s care. Although interventions are typically complex, systematic reviews often report effects according to what is thought to be the intervention’s active ingredient. The current review used Cochrane taxonomies to comprehensively determine intervention components and examined subgroups. It found that preventive primary care interventions were beneficial to older people’s functional ability and self-rated health, but positive effects were not observed for other outcomes. Future programmes should consider delivering care in alternative settings, such as home visits and phone contacts, and providing education to patients and health professionals as these may contribute to positive outcomes.

Summarising evidence with sufficient detail to guide development of intervention programmes can be particularly challenging for complex interventions. As a case in point, reviews on preventive primary care interventions that focus on multidimensional assessment have found benefits, such as reduced admissions to hospitals and nursing homes,^8^ and better functional ability.^8^^,^^9^^,^^17^ In contrast, interventions that include multidimensional assessments as an intervention component but are primarily focused on a change in the setting of care delivery (preventive home visiting) or enabling patients to improve their health (health promotion and disease prevention programmes) have been less successful in demonstrating consistent effects on healthcare use^18^^,^^19^ and functioning.^19^^,^^20^ This brings to the fore the importance of teasing out a specific intervention configuration that would most reliably yield positive health outcomes in different contexts, but previous research has found it difficult to disentangle the unique effects of potentially interacting intervention components and features.^11^^,^^15^^,^^16^^,^^18^^–^^20^ It is for this reason that the aim of the current study was to examine the overall impact of primary care interventions and to identify key intervention components that contribute to an effect on healthcare use, functional ability, and QoL.

## Method

This is a systematic review and meta-analysis of randomised controlled trials (RCTs) that examined the effectiveness of preventive interventions for older people delivered in primary care that had an impact on decreasing admissions to hospital and ARC placements, and on improving functional ability and QoL, compared with usual care.

### Study selection

A search of studies published from 2009 to 2019 was conducted in PubMed, MEDLINE, Embase, Web of Science, CENTRAL, CINAHL, and the Cochrane Library using terms that included ‘preventive’, ‘interventions’, ‘primary care’, and ‘older people’ (see Supplementary Information S1 for a full list of search terms). The current study looked at studies from 2009 onwards as a previous review on multicomponent interventions for frail older people included work until late 2008.^15^ In addition, inadequate intervention reporting is a long-standing issue,^22^^–^^26^ possibly even more so for trials pre-dating reporting tools and guidance such as the 2008 MRC framework. The authors of the current study therefore chose to exclude earlier studies as the aim was to examine specific intervention components in this review.

Titles and abstracts were independently examined by two reviewers using pre-defined eligibility criteria. The authors selected reports on intervention trials aimed at reducing admissions to hospital and ARC placement or improving functional ability and QoL, and where:

- the sample consisted mainly of people aged ≥65 years — for studies that included adults aged <65 years, a mean age of ≥70 years was required or a separate reporting of outcomes by age group;
- the intervention was delivered in primary care; and
- the study had a general focus, that is, it was applicable to the general older primary care population and was not disease specific.

Studies were excluded that:

- were not RCTs;
- focused primarily on a pharmacological or psychological intervention; or
- did not include admissions to hospital or residential care (together referred to as healthcare use), functional ability, or QoL as outcome measures.

### Data extraction

Data extraction was performed by four reviewers, with two reviewers assigned to each study included for review. Study characteristics extracted include sample population age, sample size, location of the intervention, longest duration of follow-up, description of the intervention and control groups, outcome measures, and the findings of the study. The taxonomies of two Cochrane review groups were used in coding intervention domains and components (see Supplementary Information S2).

Effects were coded as an intervention benefit where studies reported statistically significantly fewer admissions to hospital or ARC, better functioning, or higher QoL in favour of the intervention group; otherwise, they were coded as having nil effect or favouring the control. *P*-values <0.05 were considered statistically significant. Effect estimates were independently extracted from studies by the assigned reviewers.

For studies that examined healthcare use, the proportion admitted to hospital, the total number of hospital admissions, the total number of days in hospital, and the proportion admitted to ARC over the follow-up period were extracted. For studies that examined functional ability, basic and extended activities of daily living (ADLs) scores on follow-up were extracted. For studies that examined QoL, outcome measures extracted were the proportion reporting positive self-rated health (SRH), that is, those selecting response options good and above, the SRH score for studies that treated SRH as a continuous variable, QoL index scores, physical QoL domain scores, and mental QoL domain scores on follow-up.

Study investigators were contacted to obtain additional information when necessary. Estimates were transformed into odds ratios (ORs), incidence rate ratios (IRRs), ratios of durations, and standardised mean differences (SMDs), as appropriate.

### Quality assessment

Assigned reviewers used the *Cochrane Handbook for Systematic Reviews of Interventions* tools for assessing risk of bias (original tool) and quality of evidence.^27^

### Data synthesis

Data on intervention effects were synthesised narratively and through meta-analysis. Narrative synthesis of intervention effects by intervention domains and components involved vote counting based on direction of effect and statistical significance, which is further described in Supplementary Information S2. Random-effects meta-analysis was performed to obtain pooled intervention effects for similar outcome measures. To adjust for clustering inherent in the design of some trials, the authors of the current study inflated standard errors of estimates from cluster RCTs by multiplying to the square root of their respective design effects. Pooled effects were also examined according to the most common intervention components. Confidence intervals (CIs) were constructed using a 95% confidence level.

R and Microsoft Excel were used for overall management of data and analysis.

## Results

**Figure 1.**
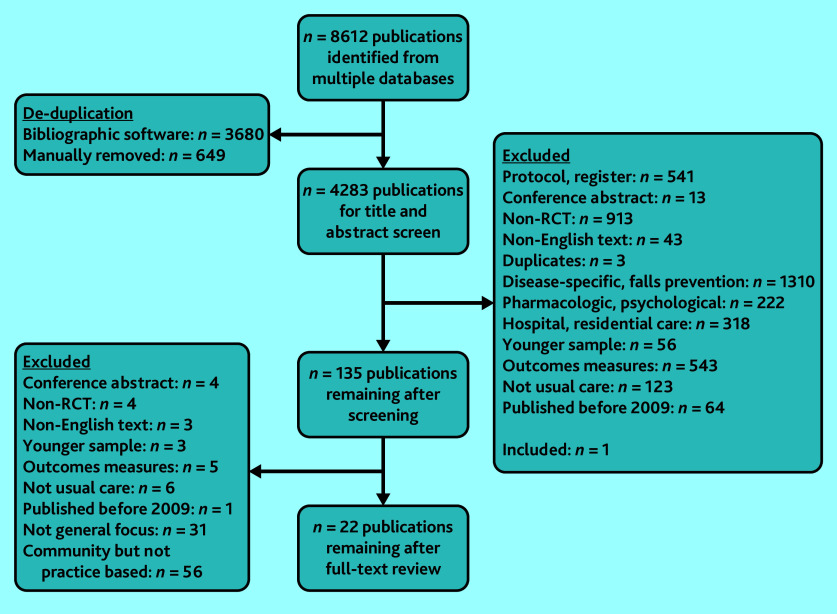
Flow diagram of the review. RCT = randomised controlled trial.

The search resulted in 8612 publications. The initial screening yielded 135 eligible publications; agreement was 97.6% (4180 of 4283 publications). Eighteen studies from 22 publications remained for data extraction after examining full reports.^28^^–^^49^ Reasons for exclusion are outlined in the flow diagram (Figure 1).

Table 1 presents the characteristics of 18 studies (total of 22 publications) included in the review.^28^^–^^49^ Most studies were conducted in Europe and North America. The median sample size of included studies was 743 (interquartile range [IQR] 267–2057), and the median duration of follow-up was 12 months (IQR 12–21). Many studies had a mix of patient-, provider-, and practice-focused intervention components (13 of 18 studies).^29^^–^^35^^,^^37^^–^^40^^,^^42^^,^^43^^,^^45^^–^^47^^,^^49^ Five studies included financial interventions such as provider incentives.^30^^,^^31^^,^^34^^,^^36^^,^^37^^,^^42^^,^^43^ None of the studies used regulatory interventions such as changes to professional licensure or medical liability. Of the three outcomes of interest for this review, QoL was more frequently reported (17 studies); the exception was Schmidt-Mende *et al*.^44^

**Table 1.** Characteristics of studies included in the review (*n* = 18)

**Characteristic**	**Studies, *n*^a^**
**Country**	
Netherlands	5
Canada	3
Sweden	2
UK	2
US	2
China	1
Germany	1
New Zealand	1
Spain	1

**Sample size, median (IQR)**	743 (267–2057)

**Months’ follow-up, median (IQR)**	12 (12–21)

**Intervention domains^b^**	
Patient	15
Provider	18
Practice	14
Financial	5
Regulation	0

**Outcomes of interest^b^**	
Healthcare use	13
Functional ability	15
Quality of life	17

a *Data are* n *unless otherwise specified.*

b *Multiple classification allowed. IQR = interquartile range.*

When Cochrane intervention taxonomies were used to code study interventions, the reviewed studies included 28 of 53 possible intervention components. Supplementary Table S1 shows the number of studies that included a specific intervention component. The most common intervention components were changes to the setting or site of service delivery such as home visits or phone contacts (14 studies); education for health professionals (14 studies); providing new clinical information to health professionals (13 studies); individual or group discussion with patients (12 studies); negotiation or decision making with patients (11 studies); and patient education (11 studies).

Overall, studies included in the review were assessed to have low-to-moderate risk of bias (see Supplementary Figure S1). Across the risk assessment domains, incomplete outcome data had the largest proportion of studies categorised as high risk. The median attrition rate of included studies was 23% (IQR 11%–35%). Most studies reported results for multiple outcomes of interest (17 of 18 studies); the exception was Schmidt-Mende *et al*.^44^

None of the studies that examined healthcare use (0 of 13 studies) and less than half of studies that examined functional ability (five of 15 studies) and QoL (six of 17 studies) reported differences favouring the intervention. When intervention effects on outcomes according to intervention domains (see Supplementary Table S2) and components (see Supplementary Table S3) were examined, differences in outcomes were generally nil or favouring the control.

### Meta-analysis of intervention effects on older people’s outcomes

**Figure 2.**
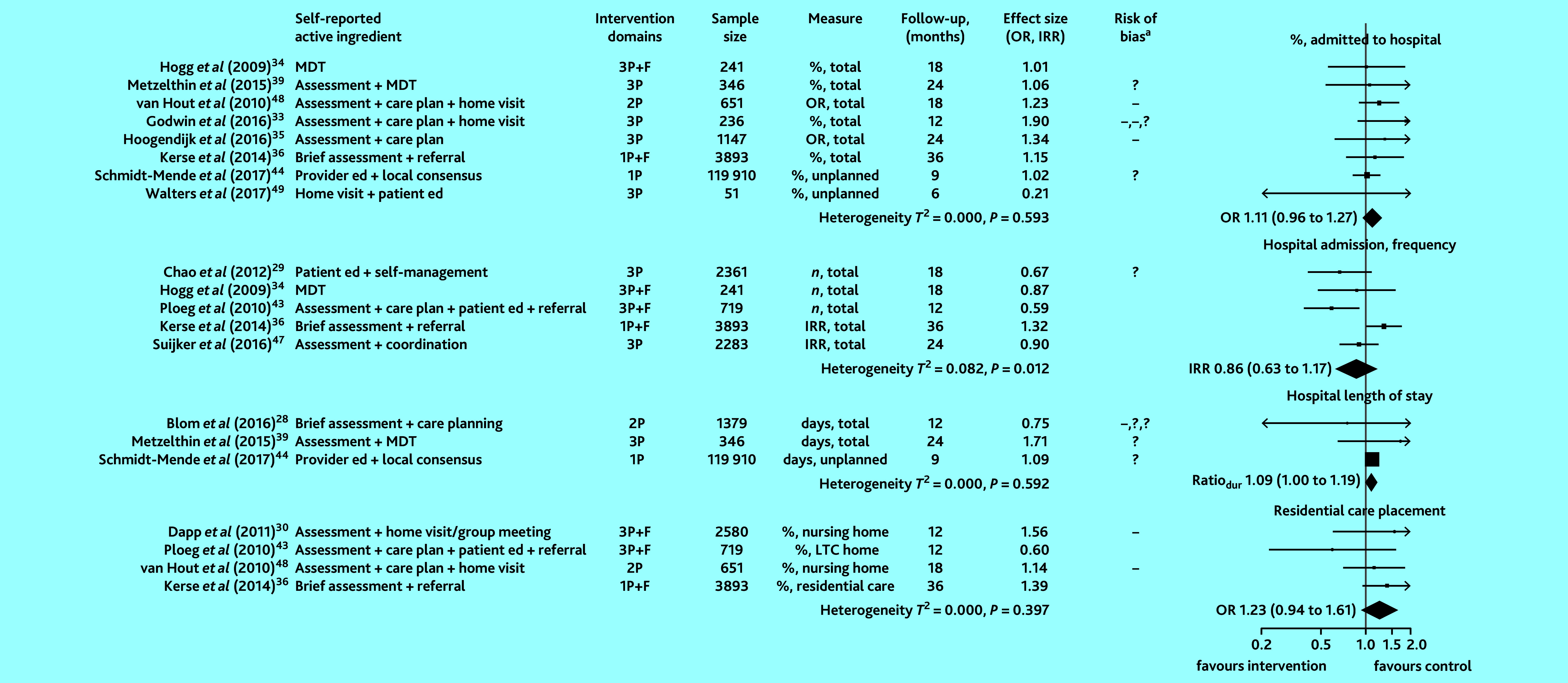
Effect of preventive primary care interventions for older people on healthcare use (*n* = 13). Data in the final column in brackets are 95% confidence intervals. ^a^High and unclear risk of bias in domains assessed are marked using the conventional style: – for high risk of bias and ? for unclear risk (>1 – or ? reflects bias in >1 domain). 1P = provider-focused domain only. 2P = provider- and practice-focused domains for van Hout *et al* (2010);^48^ provider- and patient-focused domains for Blom *et al* (2016).^28^ 3P = includes patient-, provider-, and practice-focused intervention components. %, LTC home = proportion admitted to long-term care home. dur = duration. ed = education. F = financial components. IRR = incidence rate ratio. MDT = multidisciplinary team. OR = odds ratio.

All studies that examined healthcare use as an outcome (13 of 13 studies) provided sufficient information to compute ORs, IRRs, or ratios of durations. Effect estimates in Figure 2 are grouped into the following outcome measures: proportion admitted to hospital (low-to-moderate quality evidence), frequency of hospital admissions (moderate-quality evidence), length of stay in hospital (moderate-quality evidence), and proportion admitted to ARC (moderate-to-high quality evidence). Heterogeneity in studies examining frequency of hospital admissions was statistically significant. Pooled intervention effects are represented as diamonds in Figure 2. No significant difference in measures of healthcare use were observed.

**Figure 3.**
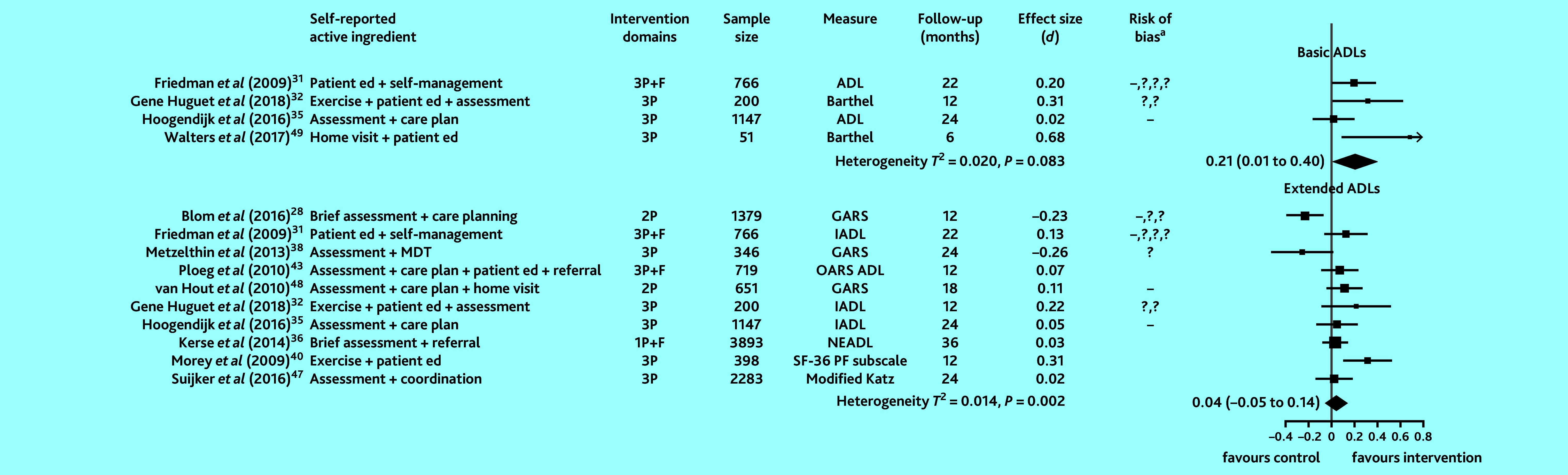
Effect of preventive primary care interventions for older people on functional ability (*n* = 11). Data in the final column in brackets are 95% confidence intervals. ^a^High and unclear risk of bias in domains assessed are marked using the conventional style: – for high risk of bias and ? for unclear risk (>1 – or ? reflects bias in >1 domain). 1P = provider-focused domain only. 2P = provider- and practice-focused domains for van Hout *et al* (2010);^48^ provider- and patient-focused domains for Blom *et al* (2016).^28^ 3P = includes patient-, provider-, and practice-focused intervention components. ADL = Activities of Daily Living. Barthel = Barthel Index of Activities of Daily Living. *d* = SMD, standardised mean difference. ed = education. F = financial components. GARS = Groningen Activity Restriction Scale. IADL = Instrumental Activities of Daily Living. MDT = multidisciplinary team. Modified Katz = modified Katz Activities of Daily Living. NEADL = Nottingham Extended Activities of Daily Living. OARS ADL = Older Americans Resources and Services Activities of Daily Living scale. SF-36 PF subscale = 36-Item Short Form Health Survey Physical Functioning subscale.

In studies that examined functional ability (15 studies), 11 provided sufficient information to compute SMDs and pool intervention effects for basic and extended ADLs. Evidence on basic ADLs was assessed to be of low-to-moderate quality and for extended ADLs of low quality. Heterogeneity in studies examining extended ADLs was statistically significant. Figure 3 shows that basic ADL scores were higher in intervention participants (combined SMD 0.21, 95% CI = 0.01 to 0.40). There was no overall benefit to extended ADLs in intervention participants compared with controls.

**Figure 4.**
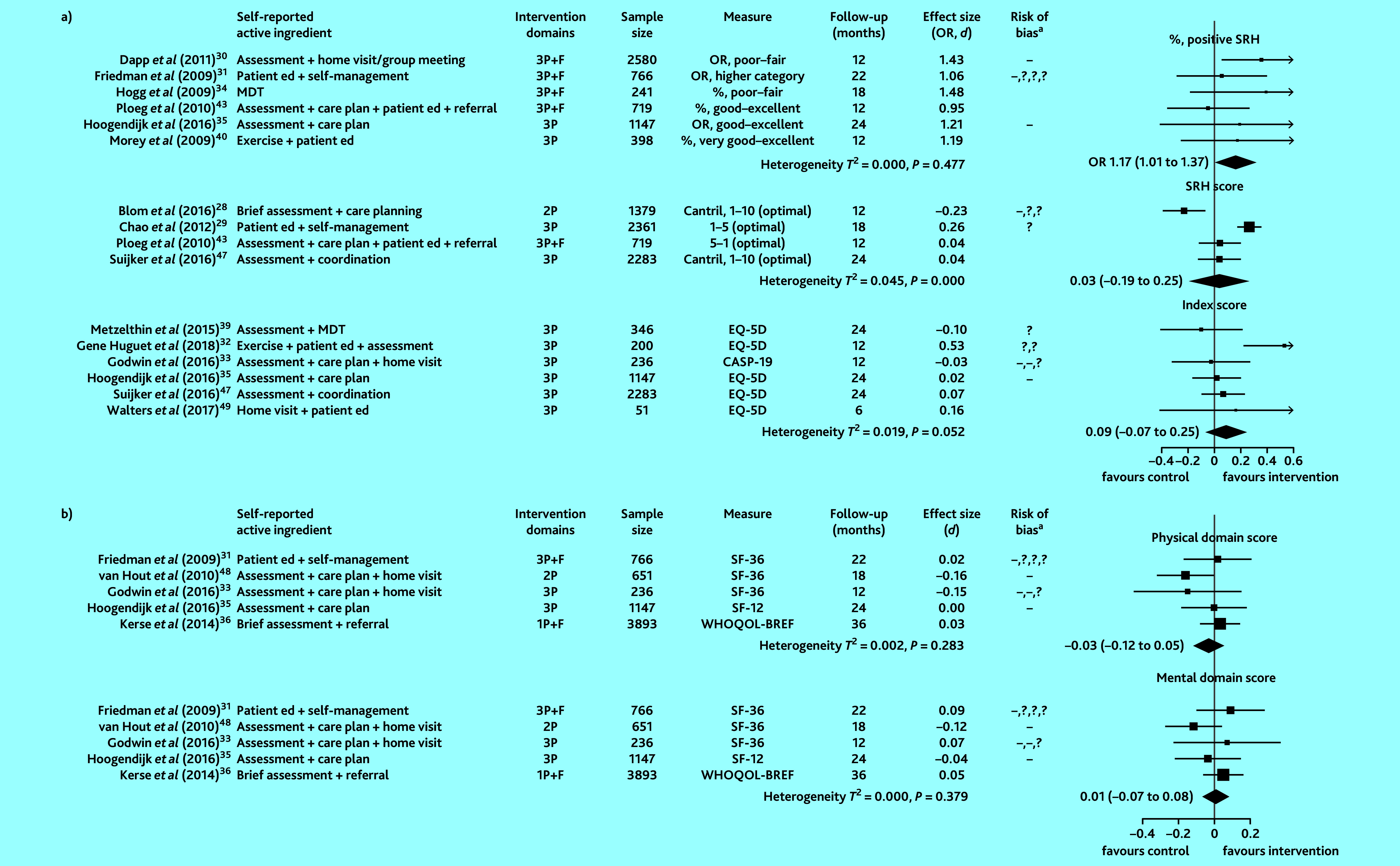
Effect of preventive primary care interventions for older people on quality of life (*n* = 15): a) single item and index scores; b) multidomain scores. Data in the final column in brackets are 95% confidence intervals. ^a^High and unclear risk of bias in domains assessed are marked using the conventional style: – for high risk of bias and ? for unclear risk (>1 – or ? reflects bias in >1 domain). 1P = provider-focused domain only. 2P = provider- and practice-focused domains for van Hout *et al* (2010);^48^ provider- and patient- focused domains for Blom *et al* (2016).^28^ 3P = includes patient-, provider-, and practice-focused intervention components. Cantril = Cantril’s Ladder. CASP-19 = Control, Autonomy, Self-Realization and Pleasure scale. *d* = SMD, standardised mean difference. ed = education. EQ-5D = EuroQOL five dimensions summary index for quality of life. F = financial components. MDT = multidisciplinary team. OR = odds ratio. SF = Short Form Health Survey. SRH = self-rated health. WHOQOL-BREF = brief version of the World Health Organization Quality of Life assessment tool. 1P = provider-focused domain only. 2P = provider- and practice-focused domains for van Hout *et al* (2010);^35^ provider- and patient-focused domains for Blom *et al* (2016).^28^ 3P = includes patient-, provider-, and practice-focused intervention components.

Fifteen of 17 studies that examined QoL provided sufficient information to compute ORs or SMDs. Pooled intervention effects for the proportion reporting a positive SRH (low-to-moderate quality evidence), SRH treated as a continuous score (moderate-to-high quality evidence), overall QoL index score (low-quality evidence), physical QoL domain score (very-low-quality evidence), and mental QoL domain score (very-low-quality evidence) are shown in Figure 4.

Heterogeneity was statistically significant in studies that treated SRH as a continuous score. Compared with controls, the odds of reporting a positive SRH was 17% higher in the intervention participants (combined OR 1.17, 95% CI = 1.01 to 1.37). An intervention benefit was not observed for the other QoL measures.

### Examination of intervention effects by common components

Table 2 summarise the effects of studies that included common intervention components. Intervention participants of studies that changed the setting of care, for example, by doing home visits or phone contacts, had 23% fewer hospital admissions (combined IRR 0.77, 95% CI = 0.63 to 0.95), higher (that is, better) basic ADL scores (combined SMD 0.21, 95% CI = 0.01 to 0.40), and higher odds of reporting a positive SRH (combined OR 1.17, 95% CI = 1.01 to 1.37). In studies that had educational components for patients, intervention participants had 26% fewer hospital admissions (combined IRR 0.74, 95% CI = 0.56 to 0.97) and were found to have higher basic ADL scores (combined SMD 0.28, 95% CI = 0.09 to 0.48).

**Table 2.** Effect of preventive primary care interventions with common practice-, provider-, and practice- and provider-focused intervention components

**Effect**	**All studies**		**Change in care setting**		**HP education**		**New info for HPs**		**Patient discussion**		**Patient negotiation**		**Patient education**	

	***n***	***d*(95% CI)^a^**	***n***	***d*(95% CI)^a^**	***n***	***d*(95% CI)^a^**	***n***	***d*(95% CI)^a^**	***n***	***d*(95% CI)^a^**	***n***	***d*(95% CI)^a^**	***n***	***d*(95% CI)^a^**
**Healthcare use**														
Per cent admitted to hospital, OR	8	1.11 (0.96 to 1.27)	6	1.21 (0.96 to 1.52)	6	1.08 (0.93 to 1.26)	5	1.24 (1.00 to 1.54)	5	1.18 (0.84 to 1.68)	5	1.18 (0.84 to 1.68)	2	0.69 (0.17 to 2.82)
Frequency of hospital admission, IRR	5	0.86 (0.63 to 1.17)	4	**0.77 (0.63 to 0.95)**	4	0.95 (0.71 to 1.29)	4	0.86 (0.59 to 1.23)	3	0.79 (0.60 to 1.03)	3	0.79 (0.60 to 1.03)	3	**0.74 (0.56 to 0.97)**
Hospital LOS, ratio of duration	3	1.09 (1.00 to 1.19)	—	—	2	1.08 (1.00 to 1.18)	2	1.42 (0.62 to 3.26)	2	1.42 (0.62 to 3.26)	2	1.42 (0.62 to 3.26)	—	—
Per cent in residential care, OR	4	1.23 (0.94 to 1.61)	3	1.08 (0.72 to 1.61)	3	1.31 (0.99 to 1.72)	4	1.23 (0.94 to 1.61)	—	—	—	—	2	0.97 (0.38 to 2.48)

**Functional ability**														
Basic ADLs	4	**0.21 (0.01 to 0.40)**	4	**0.21 (0.01 to 0.40)**	4	**0.21 (0.01 to 0.40)**	3	0.15 (−0.01 to 0.31)	3	0.18 (−0.06 to 0.42)	2	0.29 (−0.35 to 0.93)	3	**0.28 (0.09 to 0.48)**
Extended ADLs	10	0.04 (−0.05 to 0.14)	8	0.08 (−0.01 to 0.18)	7	0.03 (−0.07 to 0.13)	9	0.02 (−0.07 to 0.10)	7	0.02 (−0.12 to 0.15)	6	0.00 (−0.16 to 0.15)	6	0.09 (−0.05 to 0.22)

**Quality of life**														
Per cent reported positive SRH, OR	6	**1.17 (1.01 to 1.37)**	6	**1.17 (1.01 to 1.37)**	4	**1.27 (1.05 to 1.55)**	4	1.15 (0.94 to 1.39)	5	1.09 (0.91 to 1.31)	4	1.11 (0.89 to 1.37)	4	1.15 (0.95 to 1.38)
SRH score	4	0.03 (−0.19 to 0.25)	3	0.13 (−0.04 to 0.29)	3	0.03 (−0.27 to 0.33)	4	0.03 (−0.19 to 0.25)	3	−0.05 (−0.23 to 0.13)	3	−0.05 (−0.23 to 0.13)	3	0.13 (−0.04 to 0.29)
QoL index score	6	0.09 (−0.07 to 0.25)	6	0.09 (−0.07 to 0.25)	4	0.17 (−0.05 to 0.38)	5	0.09 (−0.09 to 0.26)	5	0.02 (−0.08 to 0.12)	5	0.02 (−0.08 to 0.12)	4	0.16 (−0.11 to 0.42)
Physical domain score	5	−0.03 (−0.12 to 0.05)	4	−0.07 (−0.17 to 0.02)	4	−0.03 (−0.12 to 0.07)	5	−0.03 (−0.12 to 0.05)	3	−0.02 (−0.14 to 0.10)	2	−0.04 (−0.20 to 0.11)	—	—
Mental domain score	5	0.01 (−0.07 to 0.08)	4	−0.02 (−0.12 to 0.08)	4	0.00 (−0.09 to 0.09)	5	0.01 (−0.07 to 0.08)	3	0.03 (−0.09 to 0.15)	2	−0.01 (−0.16 to 0.14)	—	—

a *Data are effect size d(standardised mean difference) unless otherwise indicated; all are presented with 95% confidence intervals. ADLs = activities of daily living. HP = health professional. IRR = incidence rate ratio. LOS = length of stay. OR = odds ratio. QoL = quality of life. SRH = self-rated health.*

In studies that included educational components for health professionals, intervention participants had higher basic ADL scores (combined SMD 0.21, 95% CI = 0.01 to 0.40) and higher odds of reporting a positive SRH (combined OR 1.27, 95% CI = 1.05 to 1.55) (Table 2). Studies contributing to these estimates provided education to GPs^28^^,^^30^^,^^34^^,^^44^ and nurses.^28^^,^^31^^,^^34^^–^^36^^,^^44^^,^^48^

## Discussion

### Summary

The current study sought to identify the key components of successful interventions for older people set in primary care. Overall, it was found that participants of preventive primary care interventions had better functional ability and were more likely to report positive SRH than controls. In addition to better patient-reported outcomes, fewer hospital admissions were observed in studies that changed the care setting and those that provided patient education. Studies that provided education to health professionals had some benefits to patient-reported outcomes but not healthcare use.

### Strengths and limitations

Many studies have investigated the impact of complex primary care interventions on older people’s outcomes, but attention towards identifying intervention components contributing to intervention success has been lacking.^50^^,^^51^ Previous reviews generally report effects according to what authors consider to be the intervention’s active ingredient — the ingredient identified could, understandably, differ based on trialists’ and reviewers’ background and research interests. The strength of the present review is in using the intervention taxonomies of two Cochrane review groups to determine intervention components regardless of whether they were active ingredients, and found many studies having a mix of patient-, provider-, and practice-focused intervention components.

In synthesising data, vote counting was performed based on direction of effect and statistical significance, a useful but limited approach as it does not account for the size of study samples or the magnitude of effects. However, the current study also went beyond narrative synthesis and combined effect sizes through meta-analysis, albeit there was heterogeneity in estimates for some outcomes. An attempt was made to identify how common intervention components may be contributing to positive outcomes, but subgroups of older primary care patients were not examined as a source of heterogeneity. This is a limitation of the present review as several studies targeted frail older people at risk of adverse health outcomes. It is possible that combined effects are closer to the null because of these studies, as older people at earlier stages of frailty or disability are more likely to show greater intervention benefits from preventive interventions.^52^^,^^53^

Earlier studies also tend to show greater benefits,^8^ thus the exclusion of studies published before 2009 is another important limitation. The original,^27^ rather than the updated,^54^ Cochrane tool was used for assessing risk of bias. As risk is assessed at the study level in the original tool and at the results level in the updated tool, it is possible that some results may have been assigned a higher risk of bias than warranted.^55^

It was noted that some studies described their interventions more extensively than others — it is possible that some intervention components might have been missed. It also cannot be excluded that there was publication bias, but it is reassuring that the estimates produced in the current study are based on trials with generally nil effects, that is, less than half of the studies reported an intervention benefit.

### Comparison with existing literature

The impact of complex interventions set in primary care on older people’s outcomes has been extensively researched in recent decades. Findings from previous reviews have been mixed overall on admissions to hospital,^8^^–^^13^^,^^56^ ARC placement,^8^^–^^10^^,^^12^^,^^13^^,^^18^ functional ability,^8^^,^^9^^,^^12^^–^^16^ and QoL.^11^^–^^14^^,^^18^ In the present review, an intervention benefit to older people’s SRH and functional ability was found. Poor SRH in later life is an important measure associated with adverse health outcomes such as cognitive impairment,^57^^–^^59^ frailty,^60^^–^^62^ and mortality.^58^^,^^63^^,^^64^ Older people with limitations in ADLs have a higher risk for admissions to hospital,^65^ although it remains unclear whether the functional benefit observed in the current review is clinically meaningful. The authors of the current review were unable to observe any study-level benefit for other patient-reported outcomes or for healthcare use.

The context within which these studies were conducted may provide some insight into the current findings. That is, considering improvements in usual primary care for older people^8^ and broader system-wide service delivery,^66^ it can be difficult to demonstrate success in improving outcomes. It is also possible that the scope for outcome improvement is not as large as would be expected given the current standard of routine care — over a third of the interventions were tested in countries known for their strong primary care tradition, for example, the Netherlands, the UK, and New Zealand. Questions remain whether complex interventions would yield substantial benefits in other contexts, for example, in low-and middle-income countries where the organisation and delivery of primary care has much room for improvement.

The current study found other positive outcomes when intervention effects were examined according to common intervention components that potentially improve older patients’ engagement with primary care. Previous reviews of home visiting programmes, that is, change in care settings, have found no difference in the number of people admitted^19^^,^^67^ and frequency of admissions,^68^ and mixed effects in functional ability,^19^^,^^20^^,^^67^^,^^69^^,^^70^ but in the current study it was noted that there were fewer admissions to hospital in the intervention participants of studies that provided care in an alternative setting and there were benefits to functional ability and SRH. In the current study both home visits and phone contacts were considered as alternatives to the usual clinic setting; both could increase frequency of engagement with primary care by removing the need for patients to travel to GP practices. This review was restricted to interventions that were integrated with primary care by excluding standalone interventions based in the community — this is a possible explanation for the inconsistency of the current findings with previous reviews.

Patient education was another component also associated with fewer admissions to hospitals and better functional ability in intervention participants. Improving patients’ knowledge and skills^71^^–^^73^ is a common theme in discussions about increasing levels of patient engagement, particularly for multimorbid older people. It would be interesting to know how these components, which could increase patients’ frequency and level of primary care engagement, will influence outcomes when delivered as part of community-based rather than GP practice-based complex interventions.

A previous Cochrane review has shown that clinicians’ adherence to recommended practice can be improved by providing them with educational activities^74^ such as meetings, outreach, audit, and feedback. In the present review, the authors note that having health professional education as an intervention component positively impacts on functional ability and SRH in older people. Taking a programme theory perspective,^21^ it is possible that educational interventions helped clinicians identify and address previously unmet or undermet needs. Capturing processes of care, for example, referral rates to allied health or specialist services, in future studies may help us understand how health professional-focused interventions translate to patient-reported outcomes for older people.

### Implications for research and practice

In summary, preventive primary care interventions had a benefit to older people’s functional ability and SRH but not for other outcomes. Many interventions had a mix of patient-, provider-, and practice-focused components. Based on current evidence, future programmes should consider including a change in the setting of care and educating patients and health professionals as these intervention components have the potential to reduce frequency of hospital admissions and to improve patient-reported outcomes in older people. The authors chose to examine healthcare use and broad patient-reported outcome measures for this review but capturing the experience and level of satisfaction of patients and providers, or including process indicators to evaluate intervention fidelity, may help provide further insight into intervention effects. Further discussion and consensus building on how intervention success is defined and measured is much needed. The authors of this review found it difficult to comment on clinical relevance and application without considering the contexts where these interventions were implemented or the theory of change underpinning them. Future reviews should thus endeavour to include systems and programme theory perspectives from the outset of their investigation.

## Data

The authors did not register a systematic review protocol for this study. Template data collection forms are available on request.
